# Xyloglucan Endotransglycosylase/Hydrolase Downregulation Increases *Nicotiana benthamiana* Tolerance to Tobacco Mosaic Virus Infection

**DOI:** 10.3390/ijms262211183

**Published:** 2025-11-19

**Authors:** Natalia M. Ershova, Ekaterina V. Sheshukova, Kamila A. Kamarova, Alfiya R. Alimova, Yana Y. Savchenko, Alexandra A. Antimonova, Tatiana V. Komarova

**Affiliations:** 1 Vavilov Institute of General Genetics, Russian Academy of Sciences, 119991 Moscow, Russia; ershova@vigg.ru (N.M.E.); sheshukova@vigg.ru (E.V.S.); kamila.kamarova@yandex.ru (K.A.K.); alimovaar@my.msu.ru (A.R.A.); alliemonova@yandex.ru (A.A.A.); 2 Faculty of Bioengineering and Bioinformatics, Lomonosov Moscow State University, 119991 Moscow, Russia; 3 Institute of Pharmacy, Sechenov First Moscow State Medical University, 119571 Moscow, Russia; jasavch@gmail.com; 4 Belozersky Institute of Physico-Chemical Biology, Lomonosov Moscow State University, 119991 Moscow, Russia

**Keywords:** xyloglucan endotransglycosylase/hydrolase, cell wall, tobacco mosaic virus, potato virus X, intercellular movement, antiviral defense, plant–virus interactions

## Abstract

The biological functions of the multiple members of the xyloglucan endotransglycosylase/hydrolase (XTH) protein family are rather diverse: XTHs are cell wall remodeling enzymes that participate in plant growth and development, are involved in responses to various environmental stresses and interactions with pathogenic and symbiotic microorganisms. However, XTHs’ role upon viral infection remains poorly understood. Here we identified and characterized *Nicotiana benthamiana* XTH (NbXTH) which is involved in responses to viral infection. We demonstrated that NbXTH is a positive regulator of intercellular transport. *NbXTH* suppression leads to the inhibition of tobacco mosaic virus (TMV) local spread, resulting in the increased tolerance of *N. benthamiana* plants to TMV. Therefore, NbXTH could be regarded as a susceptibility factor.

## 1. Introduction

The coordinated functioning of numerous cellular components underlies plant adaptation to stress. One of the first physical and biochemical barriers for pathogen invasion is the plant cell wall (CW) [1,2]. It also protects cells from other types of stress, e.g., mechanical stress and adverse environmental conditions. The CW contains a matrix of various molecules such as polysaccharides, proteins and pectins, and performs a wide range of important physiological functions. The CW is a dynamic structure, despite its apparent rigidity. Its flexibility and strength depend on the composition of polysaccharides; thus, CW plasticity is mediated by numerous enzymes that modify the polysaccharide–pectin matrix [3,4]. Loosening and remodeling of the cell wall are critical events during cell division and growth, cell shape change, and plant adaptation to environmental conditions in general, including response to various stresses. Currently, the plant CW is believed to form a functional network that can resist environmental stresses due to the dynamic complex of the polysaccharides in concert with glycoproteins and modifying enzymes [5,6].

Among key players responsible for CW plasticity are the members of xyloglucan endotransglycosylase/hydrolase (XTH) family, the enzymes able to cleave and reassemble xyloglucan molecules. This group of proteins represents a subfamily of glycosyl hydrolases family 16 [7]. The XTHs mainly possess either of the two enzymatic activities: xyloglucan endo-transglycosylase (XET, EC 2.4.1.207) or xyloglucan endo-hydrolase (XEH, EC 3.2.151) [8,9]. However, these functional activities were experimentally confirmed only for a few plant XTHs (reviewed in [9]). Historically, the XTH family was divided into three subfamilies [10]; however, with the growth of emerging sequencing data of various plant species, the boundaries between groups I and II became blurred [9]. At the same time, group III was divided into III-A and III-B subgroups based on the differences in the enzymatic activity of the proteins classified there. According to current data, XTHs play a central role in reducing cell wall rigidity to allow for plant cell growth during development and differentiation [4,11]. However, in recent years, a body of data has accumulated showing the involvement of these enzymes in the responses to a wide range of abiotic and biotic stresses [10,11,12,13,14,15,16]. As shown for XTHs from different plant species, these enzymes are involved in numerous physiological responses during adaptation [17,18,19,20]. Moreover, CW polysaccharides serve as a source of molecules that participate in the intercellular signaling and induce defense reactions in response to pathogen invasion [4]. When the CW is damaged, the plant initiates the synthesis of protective proteins and launches CW remodeling [21]. In contrast to bacterial and fungal pathogens, viruses do not mechanically damage the CW; this mainly explains why the CW modifications induced by pathogens are mostly regarded in a context of bacterial and fungal attack, but not viral [5]. Relatively little is known about the remodeling of the plant CW in response to viral invasion. Numerous enzymes and other CW-associated proteins affect viral intercellular transport by regulating plasmodesmata (PD) permeability [22]. For example, it was shown that, in response to infection with tobacco mosaic virus (TMV) as well as some other viruses, the level of PD callose depositions increases leading to the downregulation of PD permeability and restriction of intercellular transport of macromolecules [23]. Notably, such defense response might be counteracted and “neutralized” by viruses (for example, by TMV and potato virus X (PVX)) [24] that could activate β-1,3-glucanases, the callose-degrading enzymes [25].

In addition to regulation of PD functioning, viruses are able to affect other CW components. At present, the most detailed picture of the changes in the CW is in the potato/potato virus Y (PVY) pathosystem. It was shown that *XTH9* expression decreased during the first hours of PVY infection in resistant plants that developed the hypersensitive response (HR) [26]. In addition, infected potato plants were characterized by an increase in β-glucosidase, cellulose synthase and xyloglucan xyloglucosyltransferase (XTH-Xet5) by the 10th day after inoculation, resulting in the reinforcement of the CW [27]. In susceptible and tolerant plants, PVY systemic infection activates β-1,4-xylanase, which leads to CW softening [28]. CW loosening was shown to be accompanied by enhanced xylan deposition upon PVY infection in potato plants; moreover, PVY was reported to affect accumulation and localization of xylan-1/xyloglucan and XTH-Xet5. It was demonstrated that XTHs participate in CW reinforcement upon incompatible plant–virus interactions, while in susceptible plants, XTH activity leads to the loosening of the CW [29].

Nevertheless, the few studies on XTH involvement in plant–virus interactions available at present could not uncover the whole landscape of virus-induced CW modifications. Thus, the role of XTHs during viral infection, as well as the pattern of XTHs interactions with other stress-induced proteins, remains poorly understood.

Here we characterized *Nicotiana benthamiana* NbXTH, a member of the XTH family, aiming to understand its role in plant–virus interactions. Recently, it was shown that *NbXTH* expression correlated with the expression of the proviral cellular factor *NbKPILP* which stimulates TMV multiplication and intercellular transport in *N. benthamiana*, thus creating favorable conditions for infection development [30,31]. In the current study we identified all the *N. benthamiana* XTH-encoding sequences and performed a protein phylogenetic analysis in order to identify the XTH group that the characterized NbXTH belonged to. We experimentally confirmed the presence of an N-glycosylation site and N-linked glycan in this protein. Together with the predicted signal peptide it indicates that NbXTH passes via the endoplasmic reticulum (ER) and Golgi apparatus (GA) during maturation. Using a reverse genetics approach we demonstrated that NbXTH is a positive regulator of intercellular transport of macromolecules. In addition, we revealed NbXTH involvement in the development of the viral infection. Specifically, *NbXTH* downregulation resulted in the increased survival rate of *N. benthamiana* plants with systemic TMV infection and suppressed cell-to-cell transport of the virus. Therefore, NbXTH could be regarded as a susceptibility factor in the pathosystem studied.

## 2. Results

### 2.1. NbXTH Identification and Analysis

**Figure 1:**
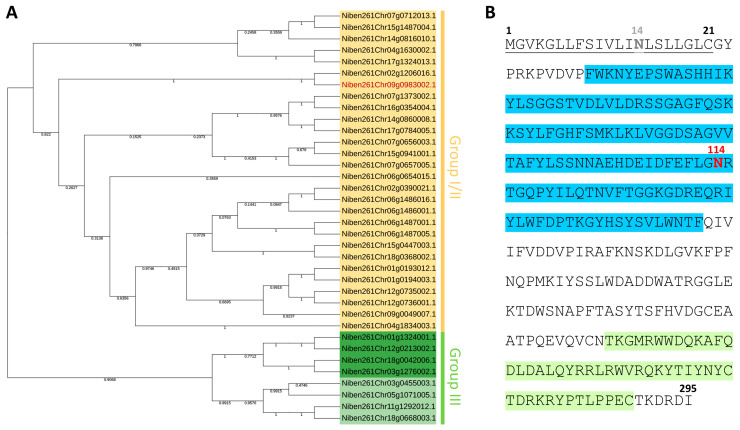
Identification and analysis of *Nicotiana benthamiana* XTHs. (**A**) Phylogenetic tree of *N. benthamiana* XTH proteins (the corresponding amino acid sequences are presented in Table S1). Composite group I/II is shown in yellow, group III-A in dark green, and group III-B in light green. The studied NbXTH protein corresponding to the identifier Niben261Chr09g0983002.1 is shown in red. (**B**) NbXTH amino acid sequence with the signal peptide underlined, PF00722 and PF06955 (Pfam) XTH protein domains highlighted in blue and light-green, and gray and red bolded “N”s stand for the N-glycosylation sites predicted with low and high probability, respectively.

The nucleotide sequence of *NbXTH* cDNA was identified using the BLAST tool and the SolGenomics database (https://solgenomics.net/, accessed on 10 April 2021). The NbXTH amino acid sequence appeared to be highly similar to *Nicotiana tabacum* endo-xyloglucan transferase (EXGT) (GeneBank Ac. BAA32518.1) (99% identity) and endoxyloglucan transferase-related protein (GeneBank Ac. BAA13163.1) (100% identity), that were designated by Wang et al. [32] as NtXTH18 and NtXTH19, respectively. We identified 36 genes encoding XTHs that putatively contained both PF00722 (glycosyl hydrolase family 16) and PF06955 (xyloglucan endo-transglycosylase (XET)) domains in the *N. benthamiana* draft genome available from SolGenomics Network (https://solgenomics.net/organism/Nicotiana_benthamiana/genome, accessed on 28 January 2025) (Table S1). Phylogenetic analysis of the XTHs allowed us to classify the studied NbXTH to the group I/II (Figure 1A). The analyzed in vitro members of this subfamily are characterized with XET enzymatic activity [9]. The similarity between the most-identified *N. benthamiana* proteins was below 50%; however, some XTHs had a high percentage identity of up to 96% (Figure S1).

According to the prediction, NbXTH contains a secretory signal peptide (Figure 1B) targeting the protein to the apoplast. To predict the presence of the N-glycosylation site, which is characteristic of the XTH proteins, bioinformatic analysis of the NbXTH amino acid sequence using the NetNGlyc service (http://www.cbs.dtu.dk/services/NetNGlyc, accessed 12 February 2024) was performed. The latter revealed the presence of two putative N-glycosylation sites, one of which was located inside the signal peptide; thus, it was not taken further into account, while another asparagine residue, Asn114, was likely glycosylated (Figure 1B).

**Figure 2:**
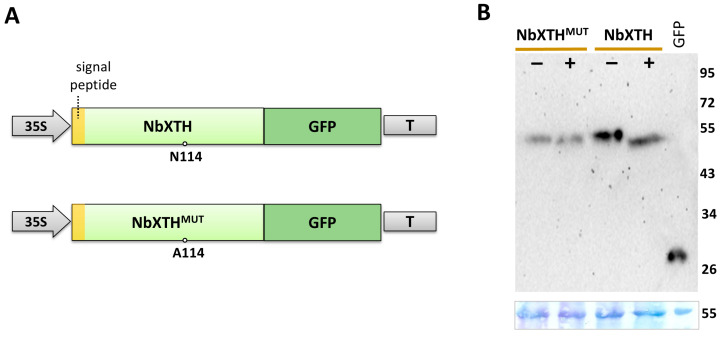
NbXTH contains N-glycosylation site. (**A**) Schematic representation of NbXTH:GFP and NbXTH^MUT^:GFP vectors. 35S: cauliflower mosaic virus (CaMV) 35S promoter; T: 35S terminator of transcription. (**B**) Western blot analysis of NbXTH- or NbXTH^MUT^-enriched preparation isolated from the leaves of *N. benthamiana* plants expressing the corresponding cassette. Samples treated with PNGase F (+) or without treatment (−) were separated in SDS polyacrylamide gel, transferred to PVDF membrane and probed with antibodies against GFP. GFP: 100 ng of recombinant GFP used as a positive control. Molecular weight markers are indicated on the right. The lower panel shows loading control: the membrane stained with amido black.

To experimentally confirm the presence of N-linked glycan we used the following approach: protein extracts from *N. benthamiana* leaves expressing the 35S-NbXTH:GFP cassette (Figure 2A) were treated with PNGase F, an amidase that cleaves the bond between the asparagine residue and the core N-acetylglucosamine (GlcNAc) of the glycan moiety. Notably, the presence of 1,3-fucose linked to that core GlcNAc prevents digestion; thus, to avoid this obstacle we used the *N. benthamiana* plant with fucosyltransferase knockdown [33]. In these plants N-linked glycans lack 1,3-fucose that allows the use of PNGase for analysis. Separation of the proteins in the SDS-polyacrylamide gel (SDS-PAAG) followed by Western blot analysis using GFP-specific antibodies revealed a clear shift of the band corresponding to NbXTH:GFP after PNGase treatment (Figure 2B). That increase in its mobility indicates that N-linked glycan was digested, which resulted in the reduction in the protein molecular weight. In addition to this approach, we constructed an NbXTH variant with Asn114 replaced with Ala (Figure 2A), expecting that NbXTH^MUT^ would lack N-linked glycan and be insensitive to PNGase treatment. Indeed, NbXTH^MUT^:GFP showed not alteration in its mobility in SDS-PAAG after PNGase treatment. This indicates the presence of only one N-linked glycan in NbXTH at position 114. Moreover, the band corresponding to NbXTH^MUT^:GFP had the same position as NbXTH:GFP after incubation with PNGase. Together, these results confirm that NbXTH is a glycoprotein containing N-linked glycan at the Asn114 residue. The presence of the signal peptide and N-linked glycan indicates that NbXTH passes via the ER and GA during maturation.

### 2.2. NbXTH Affects Intercellular Transport of Macromolecules

XTHs are known to be involved in cell wall remodeling during plant development and response to various stresses [17,18,19,20]. It could be suggested that they might affect plasmodesmata (PD) permeability and intercellular transport. To test this hypothesis, the effect of NbXTH on cell-to-cell movement of macromolecules was assessed using the 2× GFP reporter, which consists of two fused copies of GFP. Normally the 2× GFP molecule mainly remains in the cell where it was synthesized, as PD permeability for mature leaves of intact *Nicotiana benthamiana* plants is limited by the size of the protein at 47 kDa while the size of 2× GFP is 54 kDa. However, numerous cellular proteins could affect intercellular transport activity, increasing the PD aperture [34].

**Figure 3:**
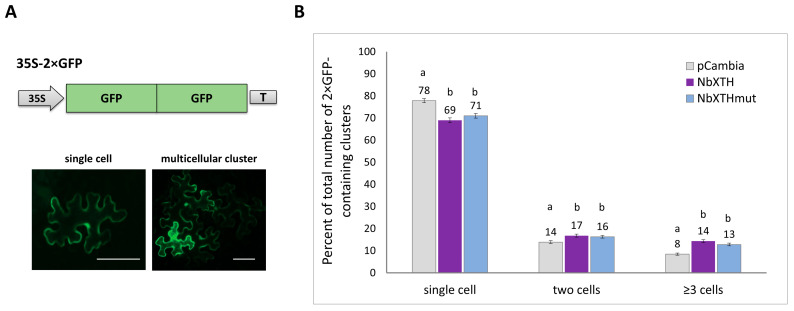
NbXTH stimulates intercellular transport of 2× GFP. (**A**) Schematic representation of the 2× GFP-encoding construct. 35S: CaMV 35S promoter; T: 35S terminator of transcription. The representative images of 2× GFP-containing single cell and multicellular clusters are shown at the lower panel. Images obtained from the fluorescent microscope. Bar = 50 µm. (**B**) Quantification of 2× GFP intercellular movement in leaves 24 h after agroinfiltration with 35S-2× GFP and 35S-NbXTH or 35S-NbXTH^MUT^. Combination of 35S-2× GFP with pCambia1300 vector was used as a control. Mean values and SE are presented. Four independent experiments with at least three biological replicates each were performed, and at least 250 cell clusters per one infiltration area were quantified. The data was analyzed using ANOVA. Bars without similar letters indicate significant differences according to Tukey’s HSD at *p* < 0.05, while bars with shared letters are not significantly different.

In this series of experiments, an optimized dilution of the *Agrobacterium tumefaciens* suspension containing the 35S-2× GFP vector was used. It allowed for delivery of the expression cassette into single cells located at a sufficient distance from each other to assess the further spread of the 2× GFP molecule to neighboring cells. Intercellular transport was assessed for three experimental groups of wild-type *N. benthamiana* leaves which were infiltrated with *Agrobacterium tumefaciens* containing the 35S-2× GFP vector in combination with (i) 35S-NbXTH, (ii) 35S-NbXTH^MUT^ or (iii) “empty” vector pCambia1300 as a control (Figure 3A). After agroinfiltration, the plants were grown under standard conditions for 24 h, after which the number and the ratio of single epidermal cells containing GFP and those grouped in clusters was analyzed (Figure 3B). The number of epidermal cells surrounding the initially transformed cell reflects the activity of intercellular transport, thus the presence of multicellular clusters indicates more efficient transport of macromolecules. It was shown that increased expression of *NbXTH* leads to activation of intercellular transport: in a group where 35S-2× GFP was co-expressed with 35S-NbXTH, the percentage of single-cell GFP-containing clusters was lower compared to the control group, while the ratio of multicellular (containing three and more cells) clusters increased (Figure 3B). Our results demonstrate that the presence of both the wild-type NbXTH and the mutant NbXTH^MUT^ proteins resulted in an increase in the observed multicellular 2× GFP-containing clusters compared to the empty binary vector. However, no statistical difference was observed between the two NbXTH variants. Thus, NbXTH N-glycosylation is not essential for its ability to affect intercellular transport.

**Figure 4:**
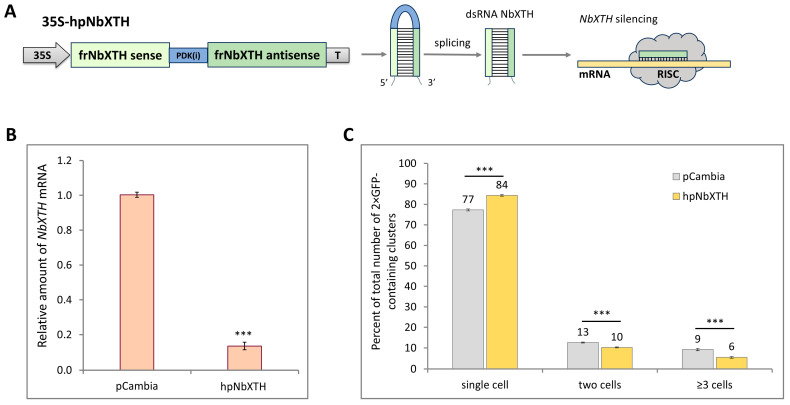
*NbXTH* downregulation negatively affects 2× GFP intercellular transport. (**A**) Schematic representation of 35S-hpNbXTH construct used for induction of *NbXTH* silencing: the synthesized RNA folds into a hairpin with an intron in the loop region. Intron processing results in the formation of a dsRNA that corresponds to the NbXTH fragment and is able to induce *NbXTH* silencing. 35S: CaMV 35S promoter; PDK(i): intron; T: 35S terminator of transcription. (**B**) Relative amount of *NbXTH* mRNA in plants agroinfiltrated with 35S-hpNbXTH or “empty” pCambia1300 vector as determined by qRT-PCR. Paired two-tailed Student’s *t*-test was applied to assess the statistical significance of the difference between plants of the two groups. ***: *p* < 0.001. (**C**) Quantification of 2× GFP intercellular movement in the presence of 35S-hpNbXTH or pCambia1300 vector. Mean values and SE are presented. Three independent experiments with at least three biological replicates each were performed, and at least 350 cell clusters per one infiltration area were analyzed. ***, *p* < 0.001 (Student’s *t*-test).

To check if *NbXTH* downregulation has a reverse effect on the intercellular transport, we used an approach based on the mechanism of RNA interference [35]. We obtained a 35S-hpNbXTH construct (Figure 4A) containing a 300-nt fragment (from 13 to 312 nt) of the *NbXTH* gene coding region in the forward (sense) and reverse (antisense) orientation with an intron separating them. Upon transcription of the construct, the synthesized RNA folds into a hairpin structure, while the intron located in the hairpin loop is excised during splicing. Thus, a dsRNA that could induce *NbXTH* silencing is produced. Indeed, 35S-hpNbXTH transient expression in *N. benthamiana* leaves led to an almost eight-fold decrease in *NbXTH* mRNA accumulation (Figure 4B), confirming efficient *NbXTH* silencing. Based on the sequence similarity analysis (Figure S2), and according to the prediction of the SGN VIGS tool, the additional potential target of RNA interference could be Niben261Chr02g1206016. To check if the corresponding mRNA is affected, we performed qRT-PCR analysis of the same samples with primers specific to this sequence. However, no RNA was detected in either control or samples from the agroinfiltrated leaves, indicating that Niben261Chr02g1206016 is not expressed in leaves (or its level of expression is negligible) and this gene is likely activated in the particular organ, tissue or development stage. Taking into account that no other sequences highly similar to *NbXTH* sequence were present among XTH-encoding genes, we could regard the studied *NbXTH* as the only RNA interference target.

Afterwards, we assessed the effect of *NbXTH* downregulation on the 2× GFP intercellular transport using the same experimental set-up as was described above. *N. benthamiana* leaves were co-infiltrated with *A. tumefaciens* containing the 35S-2× GFP vector in combination with (i) 35S-hpNbXTH or (ii) “empty” vector pCambia1300 as a control. Then, 26 h after agroinfiltration, the number and the ratio of 1-, 2-, 3-cell clusters containing 2× GFP were analyzed (Figure 4C). The percentage of single-cell clusters was higher in the group where *NbXTH* was suppressed compared to the control group, while the number of clusters containing three or more cells was lower, indicating a decrease in the activity of intercellular transport upon *NbXTH* downregulation.

Together these results demonstrate that NbXTH acts as a positive regulator of intercellular transport of macromolecules, as its increased expression led to the stimulation of 2× GFP cell-to-cell movement while decreased expression led to its suppression.

### 2.3. NbXTH Is Involved in the Development of Tobacco Mosaic Infection

**Figure 5:**
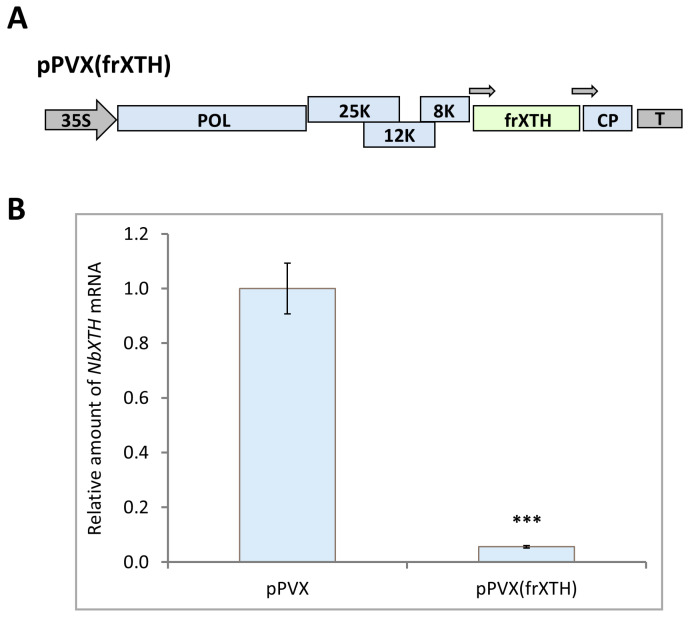
*NbXTH* virus-induced gene silencing. (**A**) Schematic representation of PVX-based vector pPVX(frXTH). Arrows indicate the PVX CP gene subgenomic promoter. 35S: CaMV 35S promoter; T: 35S terminator of transcription, POL: gene encoding PVX RNA-dependent RNA polymerase; 25K, 12K, 8K: triple gene block encoding PVX movement proteins; CP: PVX coat protein-encoding gene (**B**) Relative amount of *NbXTH* mRNA in the pPVX- or pPVX(frXTH)-infected plants as determined by qRT-PCR. The level of mRNA accumulation for pPVX-infected plants was taken as 1. Mean values and SE were obtained from four independent experiments with five biological replicates in each. The difference between samples from PVX- or pPVX(frXTH)-infected plants is significant: ***, *p* < 0.001 (paired two-tailed Student’s *t*-test).

Taking into account that (i) NbXTH was shown to be a positive regulator of intercellular transport; (ii) numerous members of the XTH family were demonstrated to participate in plant–virus interactions [26,27,29,36,37] and (iii) cell-to-cell movement is a key stage of viral infection [38], it was suggested that NbXTH may be involved in plant–virus interactions. To test this hypothesis, we first developed an experimental system for the comparison of *NbXTH* downregulation effects on the systemic infection of tobacco mosaic virus (TMV). To obtain plants with *NbXTH* suppression we used an approach based on virus-induced gene silencing (VIGS). For this we constructed a viral vector pPVX(frXTH) that encoded an infectious copy of potato virus X genome in which a 300-nt fragment (from 13 to 312 nt) of the NbXTH coding sequence was inserted under the control of the additional copy of the coat protein (CP) gene promoter (Figure 5A). *N. benthamiana* plants were inoculated with the pPVX(frXTH) vector using agroinfiltration. The pPVX vector, lacking the target gene fragment, was used as a control. Nine days post inoculation (dpi) the characteristic mild symptoms of systemic infection were observed on the upper leaves of the infected plants. Samples from those leaves were harvested and the level of *NbXTH* expression was assessed using qRT-PCR analysis. It was shown that in plants inoculated with pPVX(frXTH) *NbXTH* mRNA accumulation was 15-fold lower than in plants with pPVX systemic infection (Figure 5B). Moreover, PVX infection per se appeared to induce *NbXTH* expression compared to the mock-inoculated plants (Figure S3). Therefore, these results confirmed the efficiency of the VIGS-based approach for *NbXTH* downregulation.

For the assessment of the TMV infection development upon *NbXTH* suppression, we used the following experimental set-up (Figure S4): (i) two groups of 20 plants each were inoculated with either pPVX or pPVX(frXTH) vectors via agroinfiltration, (ii) 10–12 days later *NbXTH* downregulation in the upper leaves of the corresponding plants was confirmed by qRT-PCR analysis, (iii) plants were inoculated with TMV, and (iv) monitored for 40 days. Symptoms of TMV infection were thoroughly documented, as well as the number of the plants that survived by the end of the experiment. Thirty-five percent of plants from the “pPVX” control group survived by the end of experiment, while in the “pPVX(frXTH)” group the number of plants alive by the 40th day of monitoring was almost two-fold higher (65%). Therefore, the plants from the group with *NbXTH* silencing demonstrated a higher survival rate and tolerance to TMV infection compared to the control group (pPVX), indicating that *NbXTH* plays an important role for TMV replication and/or transport.

**Figure 6:**
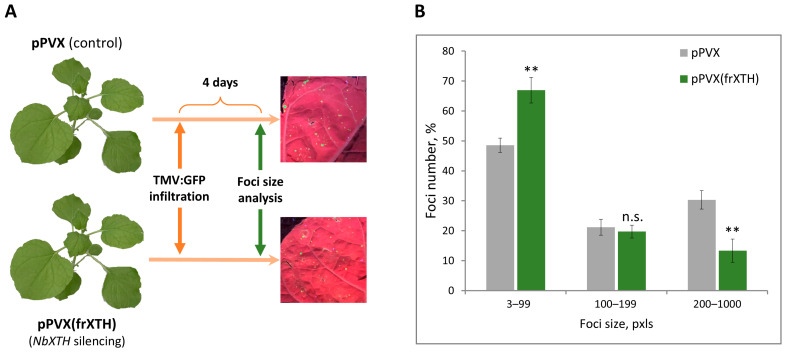
*NbXTH* silencing leads to the decrease in TMV:GFP local spread. (**A**) Schematic representation of experimental workflow: leaves of the plants from a control pPVX-inoculated group or pPVX(frXTH)-inoculated group with confirmed *NbXTH* downregulation were agroinfiltrated with TMV:GFP vector. GFP-expressing foci were visualized under UV light 4 days after agroinfiltration with TMV:GFP. (**B**) Percentage of TMV:GFP-expressing foci of different sizes. The difference between PVX- or pPVX(frXTH)-infected plants was assessed using paired two-tailed Student’s *t*-test: **, *p* < 0.001; n.s., not significant. Five biological replicates for each group were performed, no less than two areas from each infiltrated leaf were analyzed resulting in at least 1000 foci in total quantified for each group.

The success of systemic viral infection is mainly defined by local and long-distance transport. Taking into account that *NbXTH* suppression negatively affects intercellular transport of macromolecules, it could be suggested that viral local spread is also hampered by *NbXTH* silencing. To test this hypothesis, we assessed the efficiency of TMV cell-to-cell movement using a viral vector TMV:GFP, encoding the TMV genome in which the *CP* gene was replaced with *GFP*, because it allows for monitoring of TMV local spread by GFP fluorescence. This approach resulted in the initial infection of discrete cells with TMV:GFP. Upper leaves of the plants inoculated with either pPVX or pPVX(frXTH) vectors and confirmed *NbXTH* levels were agroinfiltrated with TMV:GFP (Figure 6A). TMV:GFP reproduced in the initially infected cells and moved to the neighboring cells, thus forming a focus of infection that could be visualized under UV light. The area of each focus reflects the efficiency of TMV:GFP local spread. The ratio of the foci of different sizes was assessed at 4 dpi (Figure 6B). The increase in the number of smaller foci, together with the decrease in the number of large foci in plants with *NbXTH* silencing compared to the control (pPVX) plants indicates that TMV:GFP intercellular movement is hampered by the lack of NbXTH. Therefore, NbXTH plays an important role in TMV infection, being a regulator of cell-to-cell movement.

## 3. Discussion

The plant CW is a multifunctional barrier that separates the cell from environmental threats. Secretion and targeting of multiple proteins to the CW is mediated by a conventional secretory pathway via the ER and GA. Moreover, the plant endomembrane system is essential for biosynthesis and secretion of complex CW matrix polysaccharides, glycoproteins and proteoglycans [6]. Numerous proteins undergo posttranslational modifications in the ER/GA during their maturation and then are transferred to the apoplast [39,40]. The GA is a central sorting hub of the secretory pathway; here the glycans linked to the proteins in the ER are further modified to become mature. Moreover, the synthesis of many complex polysaccharides which are subsequently incorporated into the CW is also performed in the GA [41,42]. Therefore, the GA is a major cellular compartment harboring N-linked glycan maturation and O-glycosylation of the Hyp-rich proteins and arabinogalactoproteins, in addition to de novo synthesis of such CW matrix polysaccharides as hemicelluloses and pectins [43,44,45]. The majority of the CW proteins contain glycans and reach apoplast via a conventional secretion pathway through the ER and GA [6]. Among members of the XTH family, most of the proteins are predicted to be secreted to the apoplast or anchored in the plasma membrane; however, there is evidence that not all of them go via a conventional secretion route, as was demonstrated, for example, for *A. thaliana* XTH29, which lacks signal peptide and reaches apoplast via unconventional EXPO-mediated secretion [46]. NbXTH contains a signal peptide and an N-glycosylation site, which was confirmed experimentally in the current study. This indicates that, most likely, NbXTH is transported via a conventional secretion pathway through the ER and GA, where it is glycosylated, to the apoplast; however, further localization studies are essential to confirm its exact point of destination.

We have demonstrated that enhanced *NbXTH* expression stimulated intercellular transport of macromolecules (Figure 3), likely affecting PD permeability, while *NbXTH* downregulation led to the opposite effect (Figure 4). Surprisingly, NbXTH^MUT^ lacking N-linked glycan also demonstrated the positive effect on the cell-to-cell transport of macromolecules that was not different from the effect of the native NbXTH (Figure 3). We expected a decrease in mutant variant amount and elimination of its effect on the intercellular transport. However, the level of NbXTH^MUT^:GFP was only slightly reduced (Figure 2). Previously N-glycosylation was reported to be highly important for correct folding and in vitro enzymatic activity of the PttXET16-34 from aspen [47,48] and for two of four tested XETs from *A. thaliana*. In contrast, the other two studied arabidopsis XETs were also N-glycosylated, but the removal of N-glycan did not affect their enzymatic activity [49]. Thus, we could assume that N-glycosylation is not essential for the ability of NbXTH to perform its functions, or it could be important for activities or interactions that are not involved in the regulation of the intercellular transport. Moreover, despite the proximity of the mutation (Asn114 to Ala114) in NbXTH^MUT^ to the conserved motif of the active center HDEIDFEFLG, the amino acids essential for enzymatic activity (E106, E110, D108) remained untouched. Therefore, additional experiments are to be performed to reveal the significance of NbXTH N-glycosylation.

Taking into account that cell-to-cell transport is one of the key steps defining the efficiency and success of the viral infection we checked how *NbXTH* downregulation affected TMV infection development. It appeared that *NbXTH* promoted TMV infection by stimulation of viral local spread (Figure 6). However, the mechanism underlying the involvement of NbXTH in PD permeability regulation is far from clear. There is evidence that XTHs are involved in CW strengthening in response to PVY-induced HR in potato plants, whereas in susceptible interactions these enzymes mediate CW loosening [29]. In the current study we used the compatible *N. benthamiana*/TMV pathosystem that supports TMV systemic infection that leads to chlorosis, tissue necrotization and other symptoms. Drawing an analogy with PVY-sensitive potato plants, we could hypothesize that NbXTH accumulation results in CW loosening, which in turn creates favorable conditions for TMV local spread. It is quite likely that at different stages of characteristic symptom development, *NbXTH* expression and accumulation levels vary. It can be assumed that upon appearance of the necrotic symptoms, *NbXTH* expression decreases or the protein is re-distributed from the CW inside the cell to the cytoplasm, as shown for the PVY-sensitive potato plants [29]. Such re-localization could result in CW strengthening around necrotic areas, which serves as an additional factor for restricting viral spread. And, on the other hand, in the upper systemically-infected leaves that demonstrated mild symptoms (e.g., altered pigmentation), NbXTH could localize to the CW inducing its loosening and stimulation of viral spread. Altered pigmentation indicates chloroplast dysfunctioning. Numerous chloroplast proteins are involved in organelle–nucleus–PD signaling (ONPS); thus, modulation of the corresponding gene expression affects intercellular transport, regulating the PD aperture [50,51]. It was previously shown that knockdown of genes encoding chloroplast RNA helicase ISE2, which is essential for chloroplast RNA processing and translation [52], and chloroplast RNA maturation factor PNPase [53] led to increased intercellular transport and was associated with elevated *AtXTH5* [54] or *NbXTH5* [51] expression. However, downregulation of the genes encoding two other chloroplast helicases, RH22 or RH39, also activated cell-to-cell transport but no increase in *NbXTH5* mRNA level was revealed [51]. Thus, the authors did not observe a strong correlation between *NbXTH5* expression and intercellular traffic in the experimental set-up based on the virus-induced gene silencing of the studied genes encoding chloroplast proteins. In contrast to that multifactor system, we used an approach based on monitoring of 2× GFP cell-to-cell movement upon *NbXTH5* (designated here as *NbXTH*) up- or downregulation (Figure 3 and Figure 4). Based on the obtained results we characterized *NbXTH* as a positive regulator of intercellular transport.

Our experiments demonstrated an increase in the survival rate of plants with *NbXTH* silencing upon systemic TMV infection. Moreover, in plants where *NbXTH* is downregulated we observed decreased efficiency of TMV intercellular transport (Figure 6). Previously, it was demonstrated that silencing of *NtXET-1* from *N. tabacum*, which shares 99% identity with *NbXTH*, led to reduced turnover and hydrolysis of xyloglucan, resulting in enhanced CW rigidity [55]. Thus, it could be suggested that reduction in *NbXTH* expression also results in the strengthening of the CW and, therefore, lower activity of intercellular transport. We hypothesize that *NbXTH* upregulation would have the opposite effect and lead to stimulation of TMV infection creating favorable conditions for the local and, therefore, long-distant viral transport. On the other hand, it is well-known that XTHs are involved in responses to various abiotic stresses. In particular, it was reported that another homologous (99% of identity) to NbXTH member of XTH family, NtEXGT from *N. tabacum*, mediated enhanced plant tolerance to frost and heat stresses as well as better performance upon salt stress. Moreover, *NtEXGT* expression increased in response to salinity, drought, cold and cadmium [13]. Our results demonstrate *NbXTH* upregulation upon viral infection (Figure S3). However, its increased expression leads to activation of intercellular transport, which is a key stage of successful viral infection, thus facilitating local spread of the virus. It could be suggested, that plant resistance to abiotic stresses evolutionary is more important for plant fitness and survival than resistance to such biotic stresses as viral infection, as the latter leads to the less severe consequences for the plant compared to the abiotic stresses.

Viral infections cause significant damage to agricultural crops worldwide. Discovering and characterizing the cellular factors involved in plant–virus interactions contribute to the development of virus-free agriculture. Our results show that in plants with suppressed *NbXTH* expression the efficiency TMV infection is lower than in the control plants, resulting in the increased plant survival rate. Furthermore, NbXTH stimulates intercellular transport of macromolecules, although its N-glycosylation is not crucial for performing this function. We suggest that NbXTH is a secreted glycoprotein accumulated in the CW. Thus, the increased *NbXTH* expression could result in CW loosening that, in turn, facilitates intercellular transport of the virus and the macromolecules, promoting more efficient viral infection.

## 4. Materials and Methods

### 4.1. N. benthamiana XTHs Identification and Phylogenetic Analysis

*Nicotiana benthamiana* genome and proteome were retrieved from the SolGenomics Network database (SGN, https://solgenomics.net/ accessed on 28 January 2025). To identify *N. benthamiana* XTH proteins, the following algorithm was used, as was previously reported for *N. tabacum* [32]. The Hidden Markow Model (HMM) profile of XTH protein domains PF00722 and PF06955 was obtained from the InterPro database (https://www.ebi.ac.uk/interpro/ accessed on 18 June 2025). Using the HMMER3.0 tool with the default E-value, putative XTH proteins were found. Proteins containing both PF00722 and PF06955 domains were checked for redundancy with SIAS tools (http://imed.med.ucm.es/Tools/sias.html accessed on 25 June 2025). Identification of the conserved domain of candidate XTHs was performed by the online SMART service (http://smart.embl-heidelberg.de/ accessed on 25 June 2025). After removing all redundant sequences, non-redundant sequences were kept for the further analysis.

Multiple sequence alignment of the full-length *N. benthamiana* XTH proteins was performed using MAFFT (FFT-NS-1, BLOSUM62 200PAM matrix) and UGENE 52.1 software. The phylogenetic tree was constructed using MEGA (maximum likelihood-based approach, adaptive bootstrap threshold 5.00, JTT model). Phylogenetic tree visualization was performed in the iTOL (https://itol.embl.de/ accessed on 30 June 2025).

### 4.2. Plant Growth Conditions

Wild-type *Nicotiana benthamiana* plants, as well as plants with fucosyltransferase knockdown [33] were grown in soil in a controlled environment chamber under a 16 h/8 h day/night cycle.

### 4.3. Plasmid Constructs

NbXTH coding sequence was amplified from *N. benthamiana* cDNA using the F3/R3_SalI pair of primers that introduced NcoI and SalI sites at 5′- and 3′-ends, respectively. The mutant variant of the *NbXTH* sequence was obtained as follows: first, the two fragments were amplified with the F3/R4 and F4/R3_SalI pair of primers; second, these fragments were mixed and overlap PCR was performed with F3/R3_SalI pair of primers. The resulting fragment contained NbXTH^MUT^-encoding sequence and was flanked by NcoI/SalI sites at 5′- and 3′-ends, respectively.

Either the “NbXTH” or “NbXTHmut” fragment was cloned into the pCambia-35S vector [56] using NcoI and SalI sites. Therefore, 35S-NbXTH and 35S-NbXTH^MUT^ constructs were obtained.

To obtain 35S-NbXTH:GFP and 35S-NbXTH^MUT^:GFP constructs the corresponding NbXTH sequence was amplified using the F3/R3_BamHI pair of primers and the 35S-NbXTH and 35S-NbXTH^MUT^ as a template. Each of the resulting products of PCR was digested with NcoI/BamHI and ligated together with a fragment, encoding GFP, but lacking a start codon and flanked with BamHI-SalI recognitions sites, into pCambia-35S digested with NcoI/SalI.

The plasmid 35S-hpNbXTH used for induction of *NbXTH* silencing was obtained in several steps. A 300 bp fragment of the NbXTH coding sequence (from 13 to 312 nt) was selected using the SGN VIGS tool (https://vigs.solgenomics.net/, accessed on 25 April 2023) and amplified using the F1/R1 or F2/R2 pair of primers. The first PCR product was flanked with XhoI-EcoRI recognition sites, and the second with BamHI-XbaI. The third fragment, containing the sequence of an intron from the *Flaveria trinervia* pyruvate orthophosphate dikinase (PDK)-encoding gene was obtained after digestion of a pKANNIBAL plasmid with EcoRI-BamHI restriction enzymes. All three fragments were cloned in a pKANNIBAL plasmid treated with XhoI-XbaI, resulting in an intermediate construct. At the next step, the fragment containing the 35S promoter, a 300-nt NbXTH fragment in sense orientation, a PDK intron, a 300-nt NbXTH fragment in antisense orientation and the octopine synthase gene terminator of transcription was transferred to pCambia1300 using MluI-PstI restriction enzymes resulting in 35S-hpNbXTH.

The pPVX(frXTH) vector used for *NbXTH* virus-induced gene silencing was obtained in several steps. The same 300-bp NbXTH fragment as was used for the 35S-hpNbXTH construct was amplified using the F5/R5 pair of primers, and the recognition sites of NruI and SalI restriction enzymes were introduced at the 5′- and 3′-end, respectively. PCR product was digested with NruI-SalI and inserted in the pPVX201 vector [57] under the control of an additional promoter of the CP-encoding gene, resulting in the intermediate construct. To obtain the final pPVX(frXTH) vector a fragment from the intermediate construct was transferred into PVX-BIN19 [58] via AvrII/SalI sites.

Oligonucleotides used for PCR are listed in the Table S2.

### 4.4. Agroinfiltration

*Agrobacterium tumefaciens* strain GV3101 was transformed with individual binary vectors and grown at 28 °C in LB medium supplemented with 50 mg/L rifampicin, 25 mg/L gentamycin and 50 mg/L kanamycin. Bacterial overnight culture was diluted with buffer containing 10 mM MES (pH 5.5) and 10 mM MgSO_4_, and adjusted to final OD_600_ of 0.01 (agrobacteria containing pPVX or pPVX(frXTH) plasmid), OD_600_ of 0.03 for agrobacterium carrying 35S-2× GFP plasmid, and OD_600_ of 0.3 for TMV:GFP vector. Agrobacteria, containing pCambia1300, 35S-NbXTH, 35S-NbXTH^MUT^, 35S-hpNbXTH, 35S-NbXTH:GFP or 35S-NbXTH^MUT^:GFP were diluted to the final OD_600_ of 0.1. Agroinfiltration was performed on fully expanded *N. benthamiana* leaves attached to the intact plant. A bacterial suspension was infiltrated into the leaf tissue using a 2 mL syringe. After that the plants were incubated in greenhouse conditions.

### 4.5. Protein Extract Preparation and PNGase Treatment

The samples from agroinfiltrated leaves were harvested at 3 dpi, the protein extracts were prepared as follows: 0.5–1 g of leaf material was ground to powder in liquid nitrogen followed by the addition of 3 volumes of extraction buffer (100 mM Tris, pH 8.0, 0.4 M sucrose, 10 mM KCl, 5 mM MgCl_2_, 10 mM β-mercaptoethanol and 0.1 mM PMSF). The obtained slurry was filtered through a double-layered Miracloth (Millipore, Burlington, MA, USA). The material retained on the filter was collected and washed (30–60 min incubation followed by centrifugation at 1000× *g*) 5–8 times with the extraction buffer supplemented with 0.1% Triton X-100. When the pellet lost its green color, it was washed with the extraction buffer without Triton X-100 and resuspended in one volume of 100 mM sodium-phosphate buffer (pH 7.5). PNGase F (New England Biolabs, Ipswich, MA, USA) treatment was performed according to the manufacturer’s protocol under denaturing conditions.

### 4.6. Western Blot Analysis

Aliquots from protein extracts were analyzed by SDS-polyacrylamide gel electrophoresis and blotted onto polyvinylidene difluoride membranes (GE Healthcare, Chicago, IL, USA). For NbXTH:GFP detection, the membranes were probed with polyclonal antibodies against GFP-6His raised in rabbit and conjugated with horseradish peroxidase (Evrogen, Moscow, Russia). The bands were visualized using a chemiluminescence ECL kit (GE Healthcare, Chicago, IL, USA).

### 4.7. Plant Inoculation for Systemic Infection

*N. benthamiana* plants were inoculated with pPVX or pPVX(frXTH) by agroinfiltration of the lower leaves, and in 9–11 days the systemic PVX infection was detected in the upper leaves. To induce TMV systemic infection, lower leaves of *N. benthamiana* plants were inoculated with 300 µg/mL suspension of virus particles in the presence of celite using a brush.

### 4.8. GFP Imaging and Intercellular Transport Analysis

GFP-containing cell clusters were visualized using an AxioVert 200M fluorescent microscope (Carl Zeiss, Jena, Germany) equipped with an AxioCam MRc digital camera. The excitation and detection wavelengths for GFP were 487 nm and 525 nm, respectively. The lower epidermal cells were analyzed 24-26 h after agroinfiltration with 35S-2× GFP. A minimum of 250 cell clusters per one infiltration area was analyzed and not less than three biological replicates were performed in each experimental group. Three to four experiments were performed.

### 4.9. TMV Foci Visualization and Quantification

TMV:GFP foci were visualized 4 days after infiltration using a hand-held UV lamp (λ 366 nm) and documented using a photo camera. Foci area measurement was performed using ImageJ software, version 1.47v [59] and according to the algorithm described by Zavaliev and Epel [60].

### 4.10. Quantitative Real-Time PCR (qRT-PCR) Analysis of Transcript Concentrations

Total RNA was extracted from plant tissues using the ExtractRNA reagent (Evrogen, Moscow, Russia) according to the manufacturer’s instructions. For first strand cDNA synthesis, random hexamers and oligo-dT primer were added to 2 µg of total RNA, and reverse transcription was performed using Magnus reverse transcriptase (Evrogen, Moscow, Russia) according to the manufacturer’s protocol. Quantitative real-time PCR was carried out using the iCycler iQ real-time PCR detection system (Bio-Rad, Hercules, CA, USA). The protein phosphatase 2A (PP2A) gene was used as reference gene. The target genes were detected using sequence-specific primers and Eva Green master mix (Syntol, Moscow, Russia). Primers used for qRT-PCR are listed in Table S3. Each sample was run three times, and a non-template control was added to each run. A minimum of five biological replicates were performed. The results of qRT-PCR were evaluated using the Pfaffl algorithm [61].

### 4.11. Statistical Analysis

The data was analyzed either by Student’s *t*-test or by one-way ANOVA, as indicated in figure captions. The significance of the difference between groups was assessed using Tukey’s honestly significant difference (HSD) test at *p* < 0.05 level or Student’s *t*-test. In all histograms, y-axis error bars represent the standard error of the mean values.

## Data Availability

The original contributions presented in the study are included in the article/Supplementary Materials.

## Supplementary Materials

The following supporting information can be downloaded at: https://www.mdpi.com/article/10.3390/ijms262211183/s1.
