# Supplementary material for: Xyloglucan Endotransglycosylase/Hydrolase Downregulation Increases Nicotiana benthamiana Tolerance to Tobacco Mosaic Virus Infection
Source: Int J Mol Sci. 2025 Nov 19;26(22):11183. doi: 10.3390/ijms262211183 (PMC12653722; doi:10.3390/ijms262211183)
Supplement: Supplementary file 1 [file ijms-26-11183-s001.zip › _____Ershova_Supplementary material Fig S1-S4, Table S2-S3.pdf]

Supplementary material#

#Table S1 is in the separate file

Percent Identity

1	2	3	4	5	6	7	8	9	10	11	12	13	14	15	16	17	18	19	20	21	22	23	24	25	26	27	28	29	30	31	32	33	34	35	36		
	93.8	39.2	51.4	46.6	31.4	22.1	35.2	41.6	32.6	39.8	56.9	57.6	63.5	55.3	46.7	49.8	34.7	38.8	63.9	47.2	32.8	37.5	69.7	74.1	22.9	39.6	61.0	46.5	36.1	39.6	39.4	34.5	37.3	57.7	33.5	**1**	Niben261Chr01g0193012.
		39.8	52.6	47.8	32.5	21.9	34.7	42.0	33.1	40.1	56.9	59.9	65.0	57.6	45.3	49.6	33.8	39.6	66.3	48.8	33.2	38.6	69.4	73.0	24.7	40.5	60.2	45.0	35.6	40.1	40.4	34.6	38.3	58.8	33.5	**2**	Niben261Chr01g0194003.
			35.0	37.3	37.6	39.0	36.3	30.6	39.5	27.9	39.4	41.0	40.8	31.4	35.9	38.7	30.4	35.6	39.2	40.1	39.9	96.4	38.5	39.5	22.0	36.8	40.5	35.4	31.5	37.3	37.5	35.9	68.6	39.7	38.7	**3**	Niben261Chr01g1324001.
				42.6	29.5	18.5	27.8	37.6	28.8	25.8	54.5	77.8	62.4	56.0	39.8	41.7	26.4	35.3	56.2	42.7	31.9	35.5	51.0	53.0	18.0	37.8	59.2	40.4	26.9	35.4	37.8	32.2	34.0	59.4	31.1	**4**	Niben261Chr02g0390021.
					33.3	21.8	39.9	37.4	34.3	34.9	49.3	47.5	53.1	46.1	46.5	52.4	36.1	45.4	51.9	86.0	36.8	37.5	48.0	49.3	25.9	46.2	49.1	46.3	37.1	46.2	46.8	39.5	36.1	49.1	36.2	**5**	Niben261Chr02g1206016.
						21.8	36.0	25.5	57.2	22.8	32.5	32.3	33.8	27.4	33.9	39.1	29.3	32.4	32.6	34.7	50.5	37.8	31.3	33.5	25.7	33.9	33.1	33.7	32.2	33.9	34.2	31.9	34.7	33.2	52.0	**6**	Niben261Chr03g0455003.
							24.7	19.9	21.3	28.8	21.8	23.6	24.8	25.4	25.7	28.0	23.1	26.0	22.6	24.3	22.1	42.3	23.9	24.8	17.6	25.0	22.6	26.9	23.7	25.2	26.6	19.0	53.1	23.3	21.7	**7**	Niben261Chr03g1276002.
								37.7	35.5	28.6	34.9	34.9	35.9	34.3	37.9	38.4	40.2	37.1	37.8	41.1	39.8	36.8	36.1	36.1	26.1	34.0	39.5	39.7	44.8	37.6	34.5	60.3	38.6	38.1	39.0	**8**	Niben261Chr04g1630002.
									26.7	46.1	44.0	43.5	46.3	41.5	41.4	47.4	32.7	35.0	45.3	38.7	27.2	32.1	43.0	42.5	25.9	34.4	49.6	40.5	35.1	35.5	34.0	32.7	30.7	45.3	27.8	**9**	Niben261Chr04g1834003.
										25.8	33.5	34.0	35.4	30.9	36.0	40.2	31.1	34.7	32.0	36.0	52.2	39.1	32.5	33.0	24.3	36.3	34.8	35.1	33.7	36.3	36.0	32.6	35.0	34.5	55.2	**10**	Niben261Chr05g1071005.
											34.8	38.9	42.1	40.0	38.0	42.9	36.6	32.3	36.9	36.6	24.0	28.6	37.5	37.7	30.8	33.1	40.0	39.3	38.0	31.2	32.9	25.5	30.1	41.4	24.3	**11**	Niben261Chr06g0654015.
												60.4	67.0	59.3	46.5	51.3	32.6	40.4	59.0	49.8	35.6	38.9	57.3	57.2	23.0	42.3	67.0	45.5	34.5	41.4	42.6	36.5	37.5	65.1	37.2	**12**	Niben261Chr06g1486001.
													68.5	62.4	45.5	48.4	33.9	41.6	62.4	47.8	35.3	41.1	56.5	58.8	23.7	43.3	68.3	46.6	36.2	42.7	43.1	36.3	40.2	67.7	34.1	**13**	Niben261Chr06g1486016.
														92.8	46.5	52.9	35.0	42.8	69.4	51.7	34.0	40.4	65.1	66.9	21.6	46.3	71.4	45.1	35.8	43.7	46.9	38.7	40.7	69.2	34.3	**14**	Niben261Chr06g1487001.
															40.5	48.3	25.1	37.6	64.2	45.6	30.0	30.1	58.7	61.1	25.3	39.9	65.6	38.9	27.5	37.4	40.2	31.7	32.9	63.1	30.8	**15**	Niben261Chr06g1487005.
																96.6	37.0	47.4	50.4	47.9	33.7	34.4	48.0	48.9	26.4	47.8	47.4	96.0	40.2	49.1	48.6	32.7	35.7	45.3	35.4	**16**	Niben261Chr07g0656003.
																	40.9	52.5	56.6	52.5	38.8	38.4	52.1	54.3	29.3	54.2	50.9	94.6	46.0	53.4	56.1	37.3	38.4	50.0	40.3	**17**	Niben261Chr07g0657005.
																		36.2	33.5	36.4	32.6	31.6	33.5	31.7	34.5	38.6	35.5	37.3	77.7	37.6	40.0	36.2	30.2	35.0	31.4	**18**	Niben261Chr07g0712013.
																			43.8	46.0	37.2	36.0	41.9	42.1	22.4	73.2	44.0	46.2	41.1	93.8	73.3	33.8	38.3	41.9	36.3	**19**	Niben261Chr07g1373002.
																				50.9	34.9	37.9	65.3	67.3	24.3	44.0	67.5	49.6	35.1	44.6	44.4	35.1	36.8	65.8	34.7	**20**	Niben261Chr09g0049007.
																					36.7	39.7	47.5	48.7	25.3	47.6	50.4	47.0	37.9	47.5	48.2	38.5	38.5	50.0	35.4	**21**	Niben261Chr09g0983002.
																						40.3	33.3	33.5	24.2	38.8	32.7	33.5	35.4	38.0	38.1	35.7	37.9	36.0	78.2	**22**	Niben261Chr11g1292012.
																							37.6	39.1	21.0	37.4	40.1	33.8	32.1	37.5	38.2	35.5	69.1	39.7	39.0	**23**	Niben261Chr12g0213002.
																								87.9	22.8	41.9	60.9	47.5	36.4	42.1	42.6	36.3	38.8	57.7	33.6	**24**	Niben261Chr12g0735002.
																									20.8	41.9	61.5	48.4	34.5	42.5	42.6	35.7	39.5	58.0	33.7	**25**	Niben261Chr12g0736001.
																										25.2	25.2	27.3	36.4	23.2	24.4	27.7	21.6	24.7	24.1	**26**	Niben261Chr14g0816010.
																											45.2	47.3	40.8	73.4	97.2	33.6	37.7	43.9	37.2	**27**	Niben261Chr14g0860008.
																												47.3	37.2	44.2	45.0	39.1	38.1	84.9	34.5	**28**	Niben261Chr15g0447003.
																													40.3	47.9	47.7	34.0	35.2	44.3	34.9	**29**	Niben261Chr15g0941001.
																														41.2	41.7	39.1	33.2	36.2	35.0	**30**	Niben261Chr15g1487004.
																															73.9	34.7	38.9	41.5	37.5	**31**	Niben261Chr16g0354004.
																																33.5	38.8	43.9	37.3	**32**	Niben261Chr17g0784005.
																																	37.3	38.3	34.2	**33**	Niben261Chr17g1324013.
																																		39.6	36.4	**34**	Niben261Chr18g0042006.
																																			35.1	**35**	Niben261Chr18g0368002.
																																				**36**	Niben261Chr18g0668003.

Figure S1. Identity of the amino acid sequences of *N. benthamiana* XTHs. The ID of the genes is on the right, the studied NbXTH is underlined. The full list of the corresponding sequences with their ID could be found in the Supplementary Table S1.

Percent Identity

1	2	3	4	5	6	7	8	9	10	11	12	13	14	15	16	17	18	19	20	21	22	23	24	25	26	27	28	29	30	31	32	33	34	35	36		
	52.5	55.3	62.1	55.5	56.9	50.2	47.4	71.6	66.3	49.7	50.6	51.2	45.2	93.7	45.7	61.1	43.1	39.3	47.2	52.3	44.4	61.1	63.0	47.9	67.3	52.6	44.7	72.8	50.2	55.6	46.7	63.7	44.6	61.2	46.9	1	Niben261Chr01g0193012.
		52.3	55.2	57.8	60.0	52.9	45.7	54.3	54.9	47.0	53.9	54.4	47.0	54.2	44.4	52.8	45.4	39.1	48.6	48.1	46.0	56.4	56.3	46.7	58.3	83.5	47.6	55.0	54.2	57.1	44.5	54.1	47.5	53.5	53.1	2	Niben261Chr02g1206016.
			57.1	54.7	55.2	54.7	47.0	53.9	56.1	54.5	54.7	55.1	41.8	54.9	47.2	55.0	46.3	43.5	47.1	59.1	48.3	55.4	58.1	53.7	55.6	53.9	46.7	53.5	55.1	55.8	47.0	56.8	47.8	55.1	52.4	3	Niben261Chr06g0654015.
				51.9	55.3	51.7	47.3	61.6	70.9	53.0	52.1	53.0	48.3	62.2	46.2	92.0	46.2	40.6	50.8	51.4	48.4	66.8	69.7	50.6	68.2	56.0	45.1	64.4	52.0	52.3	45.5	69.9	45.8	68.4	49.8	4	Niben261Chr06g1486016.
					93.0	53.8	47.2	55.1	52.5	52.3	53.7	57.2	45.1	54.4	44.9	51.8	47.6	40.4	49.1	50.4	48.6	54.4	56.0	49.2	56.1	56.7	47.9	53.8	56.9	95.0	45.7	53.0	48.4	52.7	53.6	5	Niben261Chr07g0656003.
						56.9	49.0	57.6	54.8	51.1	55.9	60.5	47.9	56.9	47.7	55.5	51.6	41.4	49.5	56.1	50.1	57.6	59.5	48.4	59.4	60.9	52.3	57.1	59.7	92.2	48.1	55.1	50.9	56.9	53.4	6	Niben261Chr07g0657005.
							47.7	50.2	51.9	48.0	95.3	78.7	44.7	51.1	45.4	49.7	46.6	41.6	49.1	46.8	48.2	50.5	52.2	46.9	51.5	51.8	48.5	51.3	79.0	53.7	48.8	51.2	47.3	48.6	49.2	7	Niben261Chr07g1373002.
								47.4	50.7	46.7	48.0	48.8	43.8	49.0	96.1	46.6	48.5	58.1	46.6	45.2	49.2	49.1	49.3	47.4	46.3	45.5	51.2	47.3	48.4	45.9	70.7	49.0	50.9	41.9	48.9	8	Niben261Chr12g0213002.
									64.1	50.9	50.5	53.2	46.4	70.3	46.7	60.8	46.2	43.1	49.5	53.3	46.3	61.2	66.5	49.7	67.8	55.3	44.9	86.7	51.5	54.4	49.1	62.8	45.1	64.1	50.0	9	Niben261Chr12g0735002.
										47.7	51.7	55.4	48.0	65.2	48.9	68.9	48.3	40.9	50.9	55.2	47.6	69.0	72.4	46.4	67.3	55.6	46.9	64.2	55.1	53.2	48.4	84.7	47.4	71.4	49.0	10	Niben261Chr15g0447003.
											49.3	51.6	51.5	49.5	46.9	51.9	52.2	43.3	53.2	50.0	53.0	51.1	51.8	92.6	51.7	48.3	49.2	48.9	49.8	54.4	47.4	48.1	51.1	48.0	61.3	11	Niben261Chr15g1487004.
												79.2	44.9	51.4	46.3	49.4	47.3	41.9	49.8	47.5	48.1	51.3	53.3	48.1	52.9	53.4	49.1	51.7	79.1	53.8	48.4	51.2	48.0	50.3	50.1	12	Niben261Chr16g0354004.
													45.1	52.5	47.5	51.5	45.4	42.1	50.7	51.9	48.1	54.7	56.8	51.1	54.5	55.1	49.6	54.1	97.2	56.9	47.8	54.8	48.4	52.3	52.9	13	Niben261Chr17g0784005.
														45.2	43.1	48.0	45.3	39.3	69.2	45.2	47.1	47.8	49.9	48.8	45.4	47.0	47.6	45.9	44.9	46.1	45.8	46.7	46.3	47.7	50.4	14	Niben261Chr17g1324013.
															47.0	60.8	44.0	40.4	46.5	52.4	44.7	60.9	62.8	47.7	67.7	54.7	45.9	71.7	51.7	55.1	48.1	64.4	46.2	61.9	47.0	15	Niben261Chr01g0194003.
																45.6	46.9	53.7	47.5	42.6	46.4	47.3	47.0	45.9	46.2	43.6	49.1	47.2	46.7	43.6	69.4	46.8	49.2	40.5	46.8	16	Niben261Chr01g1324001.
																	45.5	40.1	49.0	52.0	47.8	67.1	68.6	50.0	69.1	54.5	45.7	63.3	51.1	53.1	45.7	68.7	46.4	66.7	48.2	17	Niben261Chr02g0390021.
																		37.3	48.9	41.6	60.7	42.8	47.0	50.3	45.8	45.8	59.5	46.8	46.3	47.4	45.9	48.0	60.4	43.1	52.7	18	Niben261Chr03g0455003.
																			44.4	41.0	39.7	42.7	42.0	41.5	41.1	38.9	39.4	42.4	41.5	40.3	65.1	41.3	39.4	38.6	39.1	19	Niben261Chr03g1276002.
																				51.2	50.9	50.4	52.5	52.7	50.2	50.2	52.5	47.6	49.7	49.7	50.0	49.3	49.6	51.1	54.3	20	Niben261Chr04g1630002.
																					43.3	46.6	56.3	47.9	55.2	49.1	42.1	53.4	51.1	50.4	46.2	53.9	41.3	53.5	52.6	21	Niben261Chr04g1834003.
																						43.7	50.6	52.2	46.0	46.0	58.1	46.1	48.5	47.5	45.8	48.0	59.6	46.1	51.5	22	Niben261Chr05g1071005.
																							70.1	49.1	65.1	57.7	44.5	62.7	54.9	53.6	48.7	70.1	44.4	68.6	51.3	23	Niben261Chr06g1486001.
																								49.9	69.4	57.5	47.6	67.2	56.5	55.2	49.5	72.3	47.6	94.4	50.7	24	Niben261Chr06g1487001.
																									50.0	48.1	47.8	47.8	49.9	51.5	47.9	46.2	49.6	44.1	59.4	25	Niben261Chr07g0712013.
																										58.5	46.4	68.4	53.8	55.5	47.1	67.8	47.4	68.3	52.6	26	Niben261Chr09g0049007.
																											48.2	54.8	54.4	56.6	44.8	54.5	50.0	54.6	53.1	27	Niben261Chr09g0983002.
																												43.8	50.8	47.6	50.4	46.7	81.7	42.8	51.8	28	Niben261Chr11g1292012.
																													52.1	53.1	48.9	63.2	44.1	65.3	50.0	29	Niben261Chr12g0736001.
																														56.7	47.7	53.8	49.4	52.6	53.1	30	Niben261Chr14g0860008.
																															44.7	53.5	47.6	51.9	53.5	31	Niben261Chr15g0941001.
																																47.6	49.7	42.2	48.6	32	Niben261Chr18g0042006.
																																	47.3	70.7	48.3	33	Niben261Chr18g0368002.
																																		43.6	51.5	34	Niben261Chr18g0668003.
																																			46.7	35	Niben261Chr06g1487005.
																																				36	Niben261Chr14g0816010.

Figure S2. Identity of the nucleotide sequences encoding *N. benthamiana* XTHs. The ID of the genes is on the right, the studied NbXTH is underlined. The full list of the corresponding sequences with their ID could be found in the Supplementary Table S1.

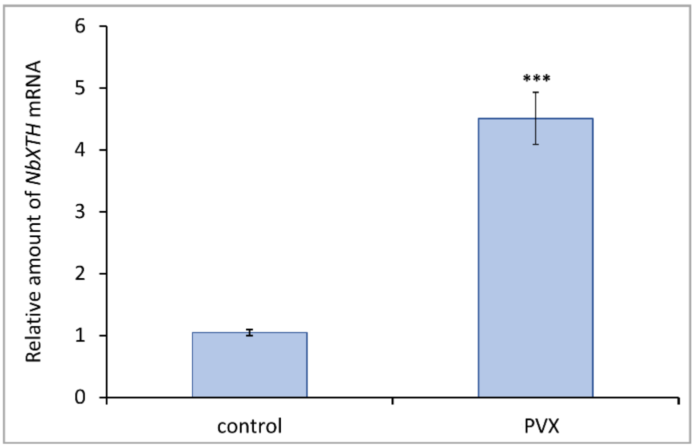

Figure S3. *NbXTH* is upregulated in response to PVX infection. Relative amount of *NbXTH* mRNA in the PVX-infected or control mock-inoculated plants as determined by RT-qPCR. Mean values and SE are presented. The level of mRNA accumulation for control plants was taken as 1. Difference between samples from PVX-infected and control plants is significant at p<0.001 (Student`s t-test), ***.

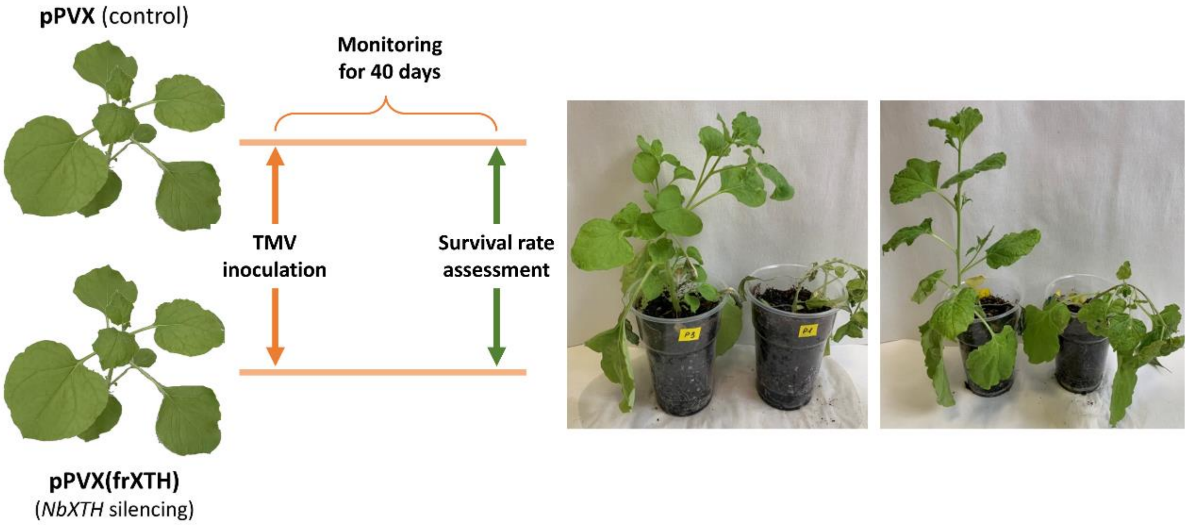

Figure S4. Systemic TMV infection in plants with *NbXTH* silencing. Schematic representation of experimental workflow: plants from a control pPVX-inoculated group or pPVX(frXTH)-inoculated group with confirmed *NbXTH* downregulation were inoculated with TMV and monitored for 40 days. The photographs of the representative plants with symptoms of TMV infection are presented.

Table S2. Oligonucleotides used for cloning

Primer	Sequence
F1	CTCGAGGGACTTTTGTTTAGTATTGTTTTGATTAATTTGTCATTAC
R1	GAATTCGTGCTCTGCATTATTCGATGACAGG
F2	GGATCCGTGCTCTGCATTATTCGATGACAGG
R2	TCTAGAGGACTTTTTTTAGTATTGTTTTGATTAATTTGTCATTAC
F3	CCATGGGTGTAAAAGGACTTTTGTTTAGTATTG
R3_SalI	GTCGACTTAAATATCCCTGTCCTTAGTGCACTCTG
R3_BamHI	GGATCCAATATCCCTGTCCTTAGTGCACTCTG
F4	CTTAGGGGCCAGGACTGG
R4	CCAGTCCTGGCCCCTAAG
F5	TCGCGAGGACTTTTGTTTAGTATTG
R5	GTCGACGTGCTCTGCATTATTCG

Table S3. Oligonucleotides used for qRT-PCR

Gene	Forward primer	Reverse primer
PP2A	ATTGCTGCCTGTGGTTATTAC	ATAGACTGAAGTGCTTGATTGG
XTH_1	ATCTTTGTGGATGACGTCCC	CATCTGCATCCCAAAGGCTC
*XTH_2**	GGCTAGTCACCACATCAAG	CTGAGTCTCCACCAACAAG
*XTH_sister***	GATCTATTCAAGCCTGTGGAG	CACTTCTTGTGGGGTGAAT

*pair of primers used for assessment of *NbXTH* mRNA level upon silencing. These primers are complementary to the region that does not overlap with a 300-nt fragment selected for silencing induction.

**pair of primers used for assessment of Niben261Chr02g1206016 mRNA level
